# A simplified microwave-based motion detector for home cage activity monitoring in mice

**DOI:** 10.1186/s13036-017-0079-y

**Published:** 2017-11-16

**Authors:** Andreas Genewsky, Daniel E. Heinz, Paul M. Kaplick, Kasyoka Kilonzo, Carsten T. Wotjak

**Affiliations:** 1 Max Planck Institute of Psychiatry, Dep. Stress Neurobiology and Neurogenetics, RG Neuronal Plasticity, Kraepelinstr. 2-10, Munich, D-80804 Germany; 2 Neuroscience Master’s Program, Interdisciplinary Center for Neurosciences (IZN), Heidelberg University, Im Neuenheimer Feld 504, Heidelberg, D-69120 Germany; 3 Department of Psychiatry and Psychotherapy, Ludwigs-Maximilians-University, Nußbaumstraße 7, Munich, D-80336 Germany; 4 Fresenius University, Infanteriestraße 11a, Munich, D-80797 Germany; 5 Institute of Applied Physiology, Ulm University, Albert-Einstein-Allee 11, N26/4406, Ulm, D-89081 Germany

**Keywords:** Home cage activity monitoring, Arduino, DIY, Open-source, Doppler-shift, Radar

## Abstract

**Background:**

Locomotor activity of rodents is an important readout to assess well-being and physical health, and is pivotal for behavioral phenotyping. Measuring homecage-activity with standard and cost-effective optical methods in mice has become difficult, as modern housing conditions (e.g. individually ventilated cages, cage enrichment) do not allow constant, unobstructed, visual access. Resolving this issue either makes greater investments necessary, especially if several experiments will be run in parallel, or is at the animals’ expense. The purpose of this study is to provide an easy, yet satisfying solution for the behavioral biologist at novice makers level.

**Results:**

We show the design, construction and validation of a simplified, low-cost, radar-based motion detector for home cage activity monitoring in mice. In addition we demonstrate that mice which have been selectively bred for low levels of anxiety-related behavior (LAB) have deficits in circadian photoentrainment compared to CD1 control animals.

**Conclusion:**

In this study we have demonstrated that our proposed low-cost microwave-based motion detector is well-suited for the study of circadian rhythms in mice.

**Electronic supplementary material:**

The online version of this article (doi:10.1186/s13036-017-0079-y) contains supplementary material, which is available to authorized users.

## Background

The monitoring of circadian rhythms in laboratory mice is essential in biomedical research and goes far beyond the issues of chronobiology. Numerous psychiatric illnesses are comorbid with sleep/circadian disturbances [1] rendering the assessment of rodent locomotor activity an important measure in pre-clinical psychiatric research. Mouse models of psychiatric disorders have been found to be ethological valid [2] with respect to changes in circadian rhythms e.g. trait-anxiety [3] and also social-stress induced depression-like behavior [4]. However, the trend to replace conventional open-top with individually-ventilated cages as well as increasing use of environmental enrichment (e.g. wooden tunnels), hinder the function of the well-proven optical based methods as visual access is obstructed. Disproportional financial expenditure for special cages and apparatuses are necessary to implement this formerly rather simple behavioral phenotyping test. Therefore it is not surprising, that the normalized numbers of publications using the term ‘locomotor activity’, ‘home cage activity’ or ‘circadian rhythm’ are regressive [5]. Conventional methods to measure home cage activity in small rodents are typically based on vibration/tilt sensing [6–8], infrared light beam crossings [9], resistance changes [10], capacitive sensing [11], video tracking [12], wheel-running [13], passive infrared emission [14], ultrasound transducers [15] but also microwave-based Doppler-shift radar systems [16–20]. Since each of these methods has its own advantages and disadvantages, we wanted our hardware to meet the following criteria: (a) flexible usage with various cage types, (b) simple, standalone operation and fast access to raw data, (c) motion detection without visual contact to the animal, (d) fast detection <1 Hz sampling rate, (e) open-source, and cost-effective, (f) easy to build with readily available tools. Here we present the design, construction and validation of a simplified microwave-based Doppler shift motion detector, with emphasis on detailed and comprehensive building instructions. Further we have applied the proposed system to assay circadian rhythmicity and photoentrainment in a mouse model which was initially established to resemble a low anxiety-related behavior (LAB) phenotype [21]. However, those animals have been found in addition to mimic certain characteristics of attention deficit hyperactivity disorder (ADHD) [22], including increased locomotor activity in emotionally challenging behavioral tasks as well as a slightly disturbed sleeping pattern [23]. We could demonstrated that LAB animals show a drastically increased homecage locomotor activity and additionally suffer from deficits in photoentrainment.

## Methods

### Animals

In this study only male LAB [21] (*N* = 3) and CD1 (*N* = 4) mice have been used. Both strains were bred in the animal facilities of the Max Planck Institute of Psychiatry, Munich, Germany. The selective breeding of LAB animals has been described extensively elsewhere [21, 22]. All animals have been single-housed >1 week prior to the experiments in Makrolon type II cages (23 × 16.5 × 14 cm) equipped with wood chop bedding and nesting material (wood wool). The animals were kept under standard housing conditions: 12h/12h inverted light-dark cycle (light off at 8 AM), temperature 24^∘^C, food and water *ad libitum*. Experimental procedures involving animals were approved (AZ 188-12) by the Committee on Animal Health and Welfare of the State of Bavaria (Regierung von Oberbayern, Munich, Germany). Animal care taking and experiments were performed in compliance with the European Economic Community (EEC) directive on the protection of animals used for scientific purposes (2010/63/EU).

### PCB design & manufacturing

The printed circuit boards (PCBs) have been designed using the cross-platform open-source electronic design automation suite KiCAD [24]. All design files are available online [25] or on request. The PCBs have been manufactured by the community printed circuit board service OSH Park [26] using the standard manufacturing parameters: two-layered FR4, 1.6 mm thickness, electroless nickel immersion gold finish, clearance >160 *μ*m, trace width >160 *μ*m, >254 *μ*m drill size. The circuit board however is rather simple (e.g. stray capacitances can be largely neglected) and a DIY solution using presensitized PCBs, UV exposure, fixation and etchants like iron(III) chloride or hydrogen peroxide/hydrochloric acid give very good results. An entire assembly using perfboard likely requires wired components, instead of the surface-mounted devices (SMD).

### Software design

The cross-platform software to write and upload the Arduino code (*see* Listing 1, [Additional file 1]) is freely available online [27]. All files (including the raw data used for this publication) are available online [25] or on request. The Python analysis script (*see* Listing 2) [Additional file 1] was written using Anaconda Python 3.5 [28]. Porting this script to Octave, MATLAB or C++ is possible with only little effort.

### Statistical analysis

All data is presented as mean values ± standard error. Statistical analysis has been performed using GraphPad Prism 5.03. One-way and two-way analysis of variance was followed by *Dunnett’s Multiple Comparison Test* or *Bonferroni* post-hoc analysis. Pearson correlation coefficients were determined using the Scipy function *pearsonr*() included the *stats* module.

## Results

### Operating principle and circuit design

**Fig. 1:**
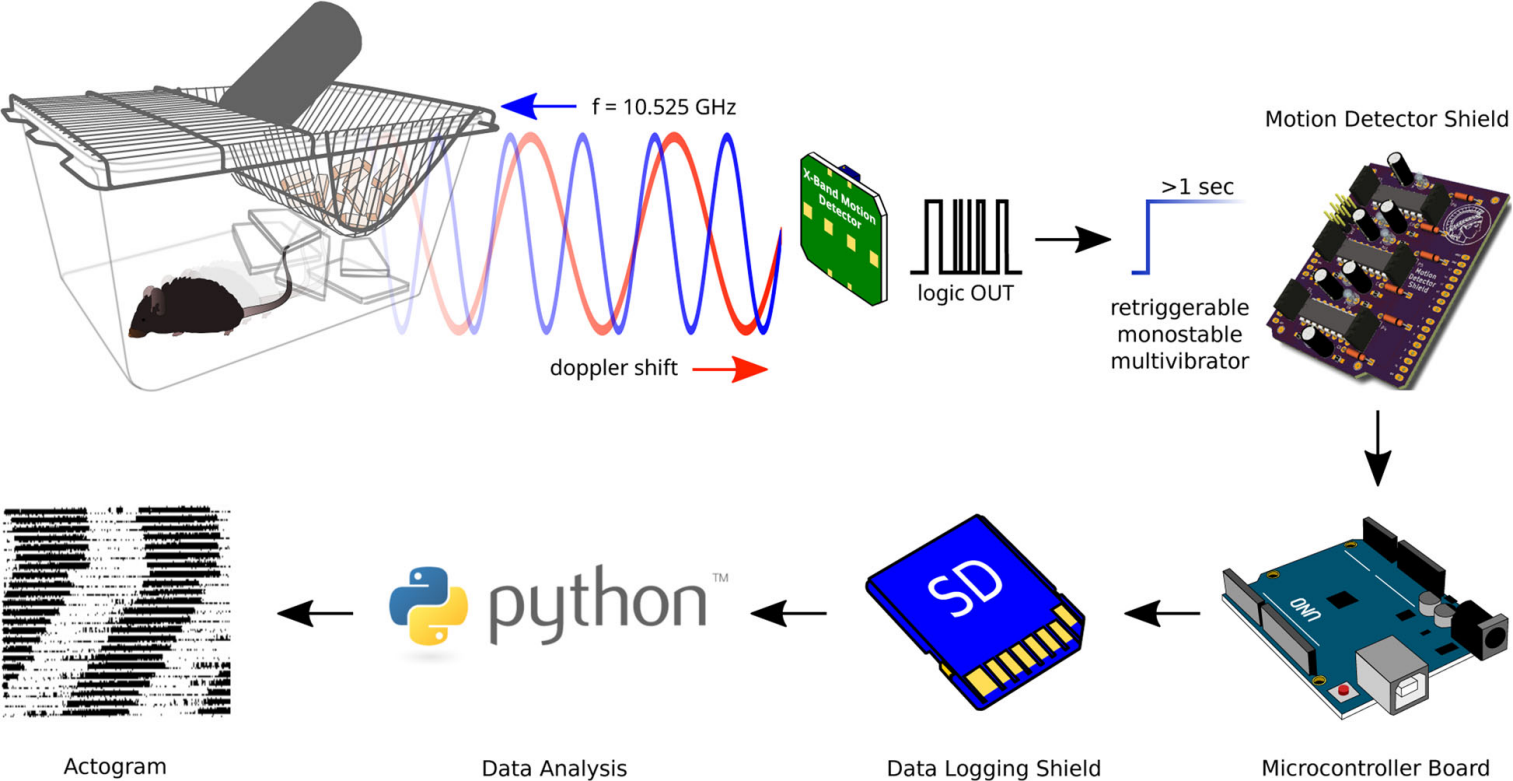
Operating Principle of the Home cage Activity Detection System. Movements of a small animal modulate and reflect the emitted 10.525 GHz radar waves via Doppler shift. This deviation from the emission frequency is sensed by the X-Band Motion Detector and an internal circuitry generates logic +5V signals according to the velocity of the animals motion. These multiple pulses of short and variable duration are transformed by the Motion Detector Shield to >1 s retriggerable pulses, and allows a downstream Arduino microcontroller board to reliably poll its I/O ports. Detected movement activity will be written to a SD card in *.csv* format with a timestamp from the realtime clock and Python scripts allow the analysis and generation of actograms

**Fig. 2:**
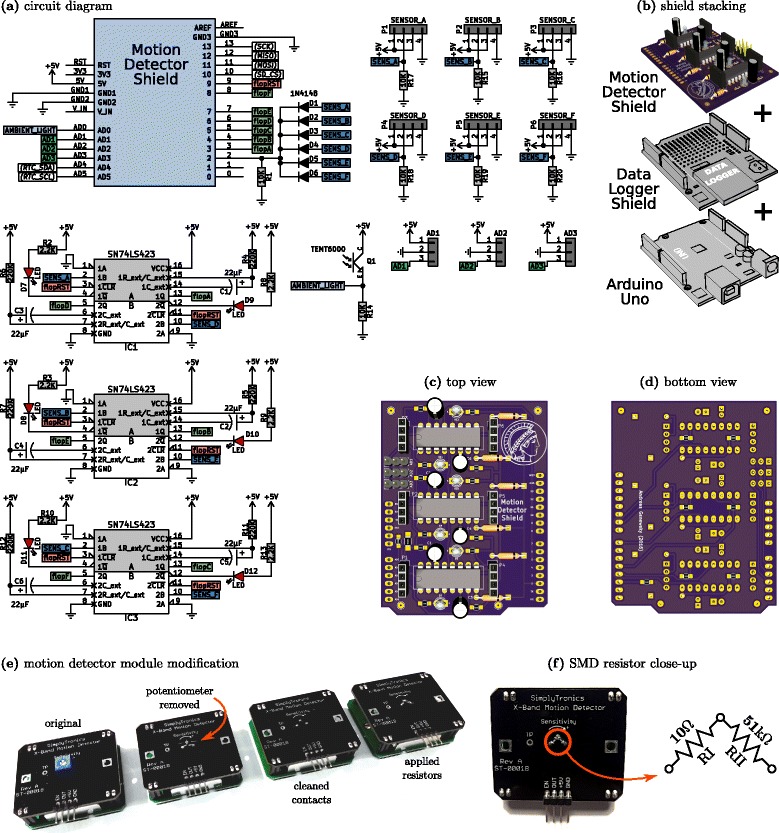
Circuit Diagram and Assembly of the Motion Detector Shield. **a** Circuit Diagram of the Motion Detector Shield (MDS). **b** The MDS is stacked onto the Data Logger Shield and ultimately both are plugged into an Arduino Uno Rev3. **c** Top view of MDS. **d** Bottom view of MDS. **e** Replacement of onboard potentiometer with SMD0805 resistor pair. **f** Detailed depiction of SMD0805 resistor placement

The overview of the operating principle of microwave-based homecage-activity monitoring system is depicted in Fig. 1. The X-Band Motion Detector module (Parallax Inc, #32213) emits electromagnetic waves at a frequency of 10.525 GHz [29], pulsed at 1.34 kHz with 25 *μ*s pulse duration (measured). These microwaves penetrate through the cage walls, bedding and housing material but are partly reflected by the animal. The frequency of the reflected waves is modulated due to the Doppler shift which allows the X-Band Motion Detector module to capture movement and output logic +5 volt signals as a function of the animals velocity. In order to reliably detect movement across up to six inputs, another interface board, the Motion Detector Shield (MDS) is necessary. Each logic output from up to six X-Band Motion Detectors is fed into three dual, monostable, retriggerable multivibrators (SN74LS423), which transform the short pulses in the microsecond range, to pulses of at least 1 s. This is sufficient for the Arduino microcontroller board to poll the digital inputs for the state. Additionally the Motion Detector Shield possesses an onboard ambient light sensor (TEMT6009) which allows to capture the light intensity. Present on the shield are three additional general purpose input/outputs pins (GPIO) or 10-bit analog-to-digital converters (ADC) in a convenient (+5V-GND-SIGNAL) three-pin configuration, which allows the easy connection of other sensors (e.g. an electret microphone) or switches. After every detected movement a short latency <5 *μ*s interrupt is generated which initiates the polling and data handling routines. After the data has been written to the SD card via the Data Logger Shield (Adafruit Industries, LLC, #1141) the multivibrators are reset, allowing the circuit to react again to new incoming movement events. The data is stored as standard comma-separated values (*.csv*) and easily accessible with various open-source spreadsheet programs like Gnumeric or LibreOffice Calc rendering our system cross-platform capable. However, simple scripts written in Python 3.5 [28] allow a quick and flexible standardized high quality analysis. The outputs from the X-Band Motion Detector modules are connected to the MDS via the pin headers P1-P6 and are summed first altogether through the diodes D1-D6 and routed to one of the Arduino’s interrupt pin. Additionally, the sensor outputs are routed to the multivibrators (IC1-IC3) where the RC circuits consisting of C1-C6 and R4-R7, R11, R12 generate >1 s pulses. These pulses are available at the Arduino’s digital inputs pin after an interrupt has been sensed by the interrupt function *detected()* (*see* Listing 1) [Additional file 1], all inputs flopA-flopF will be polled, the result will be written to the sensors[ ] array and the state variable changes (to HIGH). In the subsequent loop, the main function will see the if() condition fulfilled and writes the sensor[ ] array together with the timestamp from the RTC to the SD card. Afterwards flopRST will be pulled down briefly whereby all multivibrators are reset. Every motion event is signaled with a red (>640 nm) LED, which is only barely visible to mice and rats [30–32]. In order to completely exclude any disturbances due to the red light flashes, we recommend to cover the motion detector shield during recording (e.g. with a cardboard box) or alternatively place it outside of the recording setup. The MDS is stacked together with the Data Logger Shield onto an Arduino Uno Rev.3 microcontroller board (*see* Fig. 2 b). The components used in this design are readily available (see Table 1) and easy to hand-solder. All diodes, capacitors, connectors and integrated circuits are through-hole components, whereas the resistors are easy to handle SMD 0805 packages. The printed circuit boards were manufactured by OSH Park [26]. The design files for the MDS (*see* Fig. 2 c and e) can be directly accessed online [25] or are available upon request. The last step is to upload the code from listing 1 (*motion.ino*) [Additional file 1] in the standard way described here [33]. In order to compile the code the libraries ‘SD.h’, ‘RTClib.h’ and ‘Wire.h’ need to be installed using the Arduino IDE Library Manager. In order to use the motion detector modules in close range (e.g. type II mouse cages), we need to replace the original potentiometer with at least 50-100 k *Ω* as suggested previously [20]. The potentiometer is removed with rather ‘brute force’ using a large enough wire cutter (*see* Fig. 2 e). After cleaning the solder points with fresh solder the SMD resistors RI and RII can be applied as seen in Fig. 2 f. This leads to a rather sensitive setup and electrical shielding using aluminum foil in between the cages is necessary. Optionally the resistor RII can be replaced with 100 k *Ω*. The current consumption of the entire system (six sensors attached, all LEDs lighting up, writing data to the SD card) was maximally 180 mA and typically 135 mA, while being powered from a 12 V, 500 mA wall-wart type linear DC power supply. The current consumption of a single enabled X-Band Motion Detector module was 6.3 mA at 5.00 V. For applications were high levels of locomotor activity are expected, we therefore recommend to operate the purposed design using mains power. Another important issue is the potentially hazardous exposure to microwave radiation. According to data sheet [29] the X-Band Motion Detector modules are designed to meet the FCC rules for use within a building [34] and it is further stated that the microwave emissions are below established safety standards for general public environments [35].

**Table 1:** List of materials for the motion detector shield

Reference	Qty.	Item	Part No.	Mfr.	RS No.
AD1, AD2, AD3	3	3-pole, 2.54 mm, header	M20-9990346	Harwin	745-7068
C1, C2, C3, C4,	6	electrolytic capacitor	ECE-A1EKA220	Panasonic	807-3554
C5, C6		22 *μ*F, 25V			
D1, D2, D3,	6	1N4148, 100V, 300mA	1N4148	Fairchild Semi	843-1562
D4, D5, D6					
D7, D8, D9, D10,	6	LED, 3 mm, 1.85V, red	L-7104SRC-D	Kingbright	619-4886
D11, D12					
IC1, IC2, IC3	3	SN74LS423N	SN74LS423N	Texas Instr.	809-5661
P1, P2, P3, P4,	6	4-pole, 2.54 mm, socket	M20-7820446	Harwin	681-6814
P5, P6					
Q1	1	TEMT6000 Light Sensor	TEMT6000X01	Vishay	768-9354
R1, R14, R15,	8	10 k *Ω*, SMD 0805	CRG0805F10K	TE Connect.	223-0562
R16, R17, R18,					
R19, R20					
R2, R3, R8, R9,	6	2.2 k *Ω*, SMD 0805	CRG0805F2K2	TE Connect.	223-0477
R10, R13					
R4, R5, R6, R7,	6	220 k *Ω*, SMD 0805	CRG0805F220K	TE Connect.	223-0742
R11, R12					
–	6	X-Band Motion Detector	32213	Parallax Inc.	781-3074
		SimplyTronics			
–	6	4-pole, female, 2.54 mm	5-103960-3	TE Connect.	842-8021
–	6	4-pole, male, 2.54 mm	5-103944-3	TE Connect.	842-8093
–	1	PTFE Cable	–	RS Pro	877-5443
–	2	Arduino Stackable	PRT-11417	Sparkfun	–
		Header Kit - R3			
–	1	Data Logger Shield	1141	Adafruit	–
–	1	Arduino Uno Rev3	A000073	Arduino	769-7409
RI	6	10 *Ω*, SMD 0805	CRG0805F10R	TE Connect.	223-0152
RII	6	51 k *Ω*, SMD 0805	CRCW080551K0FKEA	Vishay	679-1525
–	1	DC power supply	8154014	RS Pro	737-8149

### Validation of the simplified microwave-based motion detector system

**Fig. 3:**
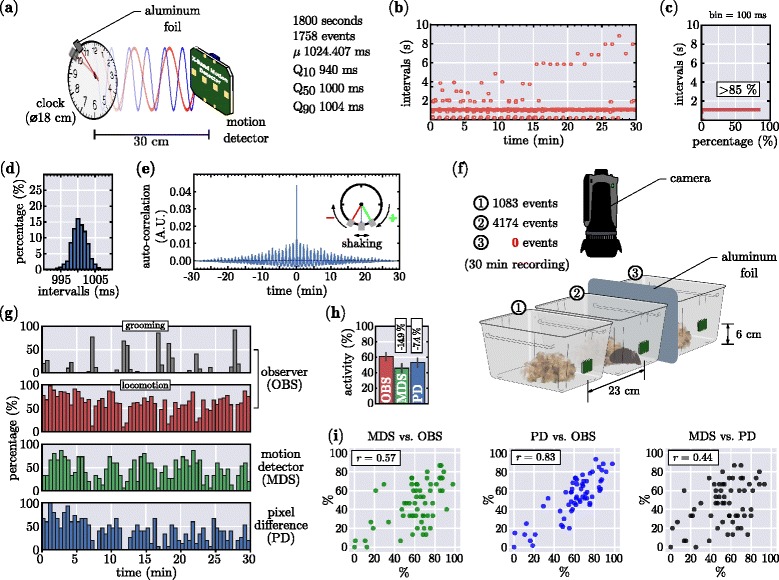
Validation of the simplified Microwave-based Motion Detector System. **a** (*left panel*) Setup for assessing the precision of the motion detector. A small piece (2 x 2 cm) of aluminum foil is mounted on the large clock-hand of an analog clock. The motion detector is placed 30 cm away from the analog clock and was allowed to capture the movements of the clock-hand for 30 min. (*right panel* Basic summary of the clock experiment; total number of detected events, mean value of event intervals and the respective percentiles are given. **b** Intervals of detected events over time. Note the occurrence of long intervals (>2 s) which indicate slight detection problems due to blind spots in the recording setup. **c** Histogram (bin = 100 ms) of all intervals demonstrating, that 85.38% of the detected events are in the range of 950 ms to 1050 ms. **d** A magnified view of the range of 990 ms to 1010 ms shows the normally distributed nature of the recorded data. **e** The auto-correlation of the recorded interval data demonstrates a prominent rhythmicity with a frequency of 1 min, which is most likely due to the additional weight on the clock-hand in combination with the slackness of the low quality gear used in the ordinary analog clock, which causes the aluminum foil to vibrate (*see inset*). **f** In oder to asses the crosstalk between simultaneously recorded, neighboring cages and the effect of shielding we have conducted another experiment (<18 lux), where one CD1 mouse was introduced to cage 2 while cages 1 & 3 where unpopulated. Further we have placed an A4-sized sheet of aluminum foil (floating, not connected to GND) between cage 2 & 3. The experiment was conducted for 30 min and in addition the behavior of the animals was video-taped. The green rectangles at the cage front show the placement of the motion detector modules. While in the unshielded cage, the detector picked up 26% of the neighboring cage, the detector of cage 3 did not detect a single event. **g** Performance comparison of the 30 min behavior (30 s bins) in cage 2 between three different locomotion detection approaches: a) an experienced observer (OBS) manually scored either the occurrence grooming (grey bars) behavior or locomotor (red bars) activity (ambulatory activity, digging and rearing); b) locomotor activity measured by the motion detector shield (MDS) (green bars); c) frame-by-frame pixel difference (PD) as an unbiased measure of movement in the video file. The pixel noise was found to generate 5.5% differences between the frames and we used a rather liberal threshold of 8.5% to determined locomotor activity. MDS and PD datasets were initially binned at 2 s bins in a binary manner (motion = 1, no motion = 0). OBS data was also binned initially at 2 s but the data was already given in percentages due to the two different variables. Further, all data set where binned to 30 s bins and the percent presence locomotion/grooming determined. **h** Averaged overall locomotor activity for OBS, MDS, PD; numbers indicate the difference to OBS. **i** Pearson correlation analysis of MDS vs. OBS, PD vs. OBS and MDS vs. PD

Besides the easy assembly and simple usage, the most important hallmark of our design is the good temporal precision. We have used an analog clock [20] with a piece (2 x 2 cm) of aluminum foil attached to the second clock hand (*see* Fig. 3 a). The motion detector was placed 30 cm away from the clock and allowed to record the clock-hand movements for 30 min (1800 s). During the recording session the system detected 1758 events with a median value of 1.000 s and an average of 1.02407 s (*see* Fig. 3 a right panel). Figure 3 b shows the intervals in between the detected motion events and notably, besides high overall accuracy, there are several intervals which fall well outside the 1 s range. These events cannot be attributed to a potential malfunction of the MDS but stem from the certainly amendable measurement setup. A quantification of the events (Fig. 3 c) reveals that 85.38% of all intervals fall into the 950-1050 ms range. A closer look at the the intervals between 990-1010 ms (Fig. 3 d) shows the normally distributed nature of the measured data. In order to test, whether our movement detection approach is qualified and sufficiently sensitive to detect rhythmic changes, we utilized the rather poor quality of the analog clockwork. An auto-correlation (Fig. 3 e) of the interval data shows the rhythmic modulation of the data every minute. This is most likely due to the additional weight on the clock-hand in combination with the slackness in the clockwork which causes the aluminum foil to shake (*see* Fig. 3 e inset). To determine the amount of shielding necessary to eliminate any crosstalk between to neighboring cages which are simultaneously measured with the MDS, we have conducted the experiment outlined in Fig. 3 f. Three cages (with bedding and nesting material) were placed close to each other, and every cage was equipped with a motion detector module (green rectangles) using double-sided tape. Between cage 2 & 3 we have introduced an A4-sized piece of aluminum foil (floating, not connected to GND) and only cage 2 contained a CD1 animal, whose activity was monitored for 30 min at dim illumination (<180 lux). While in the unshielded but unpopulated cage the motion detector picked up 26% of the neighboring cage, the aluminum foil effectively eliminated any crosstalk. In the same experiment we have in addition used a video camera to record the animals motion and an experienced observer manually scored the occurrence of grooming behavior as well as locomotion, which we defined as ambulatory activity, digging and rearing. From the video file we further deduced the frame-by-frame absolute pixel difference and used the number of changed pixels as an unbiased measure of motion in the video. This allowed a performance comparison of the three different approaches shown in Fig. 3 g. Notably both, the motion detector (MDS) and the pixel difference (PD) approach, equally reliably detect the absence of locomotor activity during high levels of grooming as well as high levels of locomotion. However, differences in the total amount of detected locomotor activity exist between the three methods (Fig. 3 h), where the PD approach resulted in 7.4% less activity compared to the human observer, the MDS approach detected 14.9% less than OBS. Pearson correlation analysis (Fig. 3 i) revealed a moderate positive correlation between the MDS and OBS (Pearsons’s *r* = 0.57), a high positive correlation between PD and OBS (Pearsons’s *r* = 0.83) and a low positive correlation between MDS and PD (Pearsons’s *r* = 0.44).

### Altered circadian photoentrainment and locomotor activity in LAB mice

**Fig. 4:**
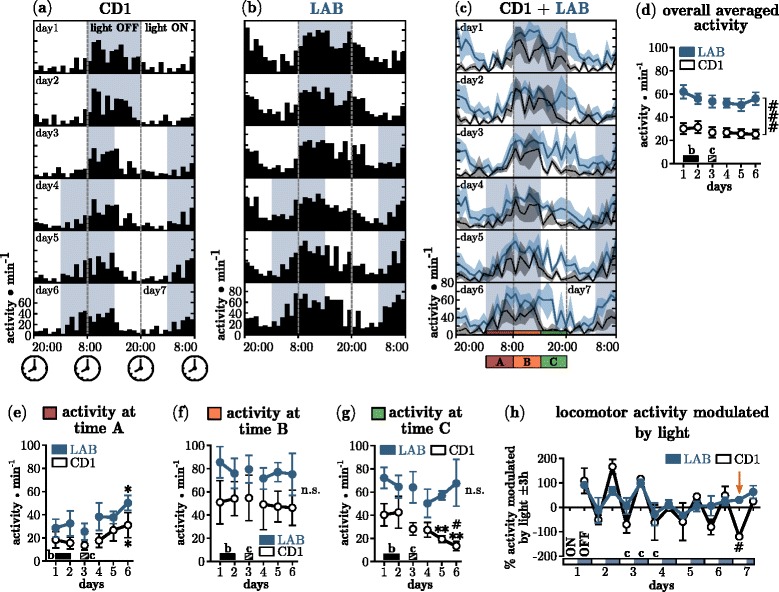
Deficient photoentrainment & and increased basal locomotor activity in LAB mice. **a** Actogram of CD1 animals (mean values only, 1h bin). White background indicates housing lights ON; gray background indicates housing lights OFF. On day 3 the light cycle was shifted -6 h, by shortening the dark period. **b** Actogram of LAB animals. **c** Overlaid actograms of CD1 (black, dashed line) and LAB (blue, solid line) with the respective SEM ranges. Colored boxes (bottom) A red, B orange, C green, indicate the three different time points which have been analyzed separately (*see* **e**–**g**). **d** Overall averaged activity of CD1 (black) and LAB (blue) mice. Inset *b* indicates the baseline recording; *c* indicates the light cycle change. **e** Activity at time point A (2 AM – 8 AM), part of the dark period after day 3. **f** Activity at time point B (8 AM – 2 PM). **g** Activity at time point C (2 PM – 8 PM), part of the light period after day 3. **h** Modulation of locomotor activity by changes in light cycle. The individual activity of the first 3h after a light cycle change, was normalized to the activity 3h before the change in order to dissect the % modulation. The arrow indicates the absence of any modulation in LAB animals. Asterisks (*) indicates 1-way ANOVA, Dunnett’s Multiple Comparison Test compared to averaged baseline: ** *p* = <0.01. Hash (#) indicates 2-way ANOVA significance values as either strain (bracket) or time differences with Bonferroni post-hoc: # *p* = <0.05, ### *p* = <0.0001. Day 3 (light cycle change, indicated by ’c’) has been excluded from statistical analysis

In order to test whether our system is able to detect the activity changes in two different mouse lines, we have made use of the low-anxiety related behaving animals (LAB mice) which were also described as a model for attention deficit hyperactivity disorder (ADHD) and were shown previously to display increased activity in emotionally challenging behavioral tasks [21, 22]. Before the onset of the experiment, the animals were kept at an inverted 12h/12h light-dark cycle (8:00PM light ON, 8:00AM light OFF) for 1 week, which was shifted minus 6h (advance) at day 3. The photoentrainment of the circadian rhythm allows the animals to adjust slowly to the new light cycle. Figure 4 a and b shows the actograms as averaged and binned (bin = 1h) homecage activity of CD1 and LAB animals. Figure 4 c depicts the overlaid activity of CD1 and LAB animals over entire course of the experiment. CD1 animals showed a pronounced circadian rhythmicity in their locomotor activity during baseline (days 1+2). By far the greatest portion of activity is observed during the dark phase. With the onset of the light phase this activity ceased. LAB animals on the contrary showed a less contrasted activity profile with high activity at the beginning of the light phase. The overall averaged activity per day (Fig. 4 d) of LAB animals was approx. two-times increased compared to CD1 controls and different throughout the experiment (*F*_1,184_ = 24.95, *p* <0.0001), confirming the hyperactivity phenotype of LAB animals. The light cycle shift (LCS) on day 3 forced the animals to adapt their activity pattern to the new onset of the dark phase. Over the course of days 4-6, both CD1 and LAB animals significantly increased their locomotor activity during the first 6h of the dark phase (Fig. 4 e) compared to baseline (CD1: *F*_2,6_ = 5.47, *p* = 0.0375; LAB: *F*_2,6_ = 5.92, *p* = 0.0317), indicating that both strains were able to adjust their circadian locomotor activity. Between 8:00AM and 2:00PM (time point B, unaltered light condition) both strains showed equal amounts of activity (*F*_1,20_ = 1.85, *p* = 0.2459), which was also unaffected by the LCS (Fig. 4 f). The locomotor activity at time point C (2:00PM to 8:00PM, now light phase) of CD1 animals at days 5+6 decreased strongly (*F*_2,6_ = 13.21, *p* = 0.0047) indicating a robust photoentraining effect (Fig. 4 g). LAB animals on the other hand did not react to the altered light cycle. The modulation of locomotion by light cycle changes became evident when the individual, averaged activity of 3h after a change, was normalized to 3h before and was plotted for every change in cycle (Fig. 4 h). During days 1-3 this modulation was prominent for CD1 and LAB mice. After the light cycle shift the modulation was severely disturbed in both strains. While LAB animals seemed to be unable to establish normal rhythmicity in the observed time window, CD1 animals could recover quickly. A strong time effect was revealed by 2-way ANOVA (*F*_9,40_ = 50.45, *p* < 0.0001) with moderate interaction (*F*_9,40_ = 2.53, *p* < 0.0212). This indicates that LAB animals are impaired in using photoentraining signals to adjust their circadian rhythm compared to CD1 controls.

## Discussion

Here we have described the design, construction and validation of a simplified microwave-based motion detector for home cage activity monitoring in mice. We have emphasized all necessary steps to copy and built the proposed project. Particular care was taken to use readily available parts in order to ease the straightforward adoption and ‘jump-start’ the application in the laboratory. We have demonstrated the high detection accuracy and temporal resolution. Moreover, we could show for the first time that animals which were selectively bred for low-anxiety behavior (LAB), a model organism for extremely low levels of trait anxiety [21] and attention-deficit hyperactivity disorder [22], have strong deficits in photoentrainment.

### Deficient photoentrainment in LAB mice

Photoentraining signals reach the retinal ganglion cells (RGC), which constitute the optic tract, and in turn send the photic information from the retina via the retinohypothalamic tract (RHT) to the two primary targets of the circadian regulatory system, namely the suprachiasmatic nucleus (SCN) and the intergeniculate leaflet of the the thalamus (IGL) [36]. However, the SCN is considered to act as the ‘master clock’ [37]. Only the photic signals perceived via the eyes carry entraining information, as binocular enucleation completely abolishes photoentrainment [38]. Theories about extraocular photoreception [39] e.g. humoral phototransduction have not been substantiated nor accepted so far [40]. Despite the photoreceptor cells within the retina (rods & cones) also as special form of RGCs have been found to be intrinsically photosensitive (ipRGC) through their photopigment melanopsin. Genetic ablation of theses cells was found to abolish photoentrainment [41, 42] and indicates that visual information via rods and cones is not necessary for functional circadian photoentrainment. This is further substantiated by studies with mice which carry a homozygous mutation in the gene *Pde6b* encoding for the rod-specific phosphodiesterase 6b. These *rd1* mice (retinal degeneration 1) loose their rod photoreceptors within the first weeks after birth, followed by a secondary, slower degeneration of the cone photoreceptor cells [43], leading to complete retinal blindness while RGC function is unaffected. These animals, however, have been shown to exhibit normal circadian rhythms [44]. The LAB mouse line descended from the commonly used outbred strain CD1 which has been described of possessing high incidences of retinal degeneration [45]. Whether LAB animals carry the *rd1* mutation is currently not known, but would otherwise also not explain their deficient circadian photoentrainment. Interestingly there is growing evidence that links ADHD with disturbed sleeping patterns and distorted circadian rhythms [46, 47]. The underlying neurophysiological changes in LAB animals, impairing the ability to entrain their circadian rhythm to photic stimuli, cannot be resolved at this stage. But a potentially altered functionality of the SCN in LAB animals could explain the hyperactivity as well as the previously described altered sleeping patterns [23] and possibly also the impairment in photoentrainment.

### Significance of the current design

Several studies so far have proposed elegant ways to monitor activity in small animals like mice and invertebrates. The by far most widely applied methods usually utilize some sort of optical readout, be it (active) infrared light beam crossings [9, 48, 49] or motion detection using passive infrared (PIR) sensors [14], which detect black body radiation in the mid infrared (≈ 3 *μ*m) range. These methods are readily applied as their operating principles are easy to comprehend and their technical implementation is rather simple. However all optical based approaches have in common that a constant, unobstructed, visual access must be guaranteed throughout the experiment, and those typically last several days up to weeks. It is therefore desirable to house the animals in their accustomed environment also during home cage activity monitoring in order to minimize distress and long acclimatization periods. With the sanitary and technical advances in animal husbandry, the conventional grid top cages are progressively replaced by individually ventilated cages (IVCs), which are typically operated in specific high-density racks. These systems provide only little space around the cages and obscure most sides especially the top side (filter top). Therefore the usage of microwave based radar systems, which have been beautifully described previously [20, 50, 51], is advisable. However, none of the previously published studies give detailed building instructions which would be necessary to enable an electronics novice to copy and apply the method. Our design is simple to implement and involves the crucial basic building blocks, like the popular Arduino microcontroller platform and the powerful scientific programming language Python, which form the core of many open-source research equipment projects [52–54]. The decision to favor the Arduino Uno microcontroller board over other devices like the single-board computers Raspberry Pi or the BeagleBone (for a comparison of the different systems see Leccese et al. 2014 [55]) was motivated by the fact that the Arduino platform is an ideal candidate for beginners due to the plethora of available online documentation, while offering more than sufficient peripherals, on-board connectivity features and computing power to fulfill the respective tasks.

There are some additional features which might be desirable to implement in the future which will be briefly mentioned: (**1**) The measured output is given as activity per minute which is simply the detector activation per minute and an ongoing locomotion triggers the detector several times. For our purpose, this measure was sufficient, but more biologically relevant measures like percent activity over time can be implemented in the Python script. (**2**) More advanced analysis parameters like period, phase and phase-shift can be obtained from the acquired data and the reader is advised to follow the excellent protocols and guidelines for analyzing locomotor activity rhythms published by Rosato et al. (2006) [56] and Jud et al. (2005) [57]. (**3**) Instead of storing the data onto a SD card, it is rather simply possible to use an additional WiFi or Ethernet shield to send the data directly to a central network storage or cloud service. Thereby, also the parallel use of several motion detectors at once is realized best. (**4**) In addition one could equip the microwave based motion detector system with one or several small serial cameras. Thereby, the entire system can be used to e.g. study wildlife animal densities in the field. The Doppler shift sensors consume very little amount of current (6.3 mA) while providing large spatial coverage. A detected motion could be used to wake up the Arduino board from deep sleep, whereby the overall power consumption is minimized enabling even battery powered operation in a reasonable manner.

### Potentially hazardous effects of microwave radiation

The FCC rules for the use of radio frequency devices within a building [34] and the established safety standards for general public environments [35] are only valid with respect to the human physiology. It is therefore an important question whether our device, emitting 10.525 GHz, might exert any biological effects on mice. First we try to estimate the emitted power of the microwave radiation. In the data sheet [29] we find the maximal effective isotropic radiated power (EIRP) of 14 dBm which is equivalent to 25.12 mW radiated power during continuous wave (CW) operation. Therefore we can predict the power density *S* ($\frac {W}{m^{2}}$) using the formula $S=\frac {EIRP(W)}{4\pi R^{2}}$ [58], where *R* is the distance in meter. This formula over-predicts the power densities in the near-field [58], but can be used to for making a ‘worst-case’ or conservative prediction. A mouse is exposed most to the microwaves, if it would build its nest directly in front of the sensor. In our experiments we have mounted the sensor modules 6 cm above the cage floor with double-sided tape directly at the outside of the cages. Considering bedding material and the approximate size of the murine body we therefore assume a minimal distance of 2 cm to the sensor. Given this distance we can estimate a power density of ≈ 0.5 mW/cm^2^. However our module does not emit this power constantly but only at less than 4% of the time, giving an approximate averaged power density of < 0.02 mW/cm^2^.

However there is compelling evidence [59] that microwave (10 Ghz) exposure to infant mice (postnatal day) at a power density of 0.25 mW/cm^2^ for 2 h/day (CW), for 15 consecutive days stresses the animals, as shown by a decreased weight gain and ultimately leads to a decreased performance in a spatial memory task (Morris water maze) later in their live (>6 weeks). Further, 10 Ghz exposure to adolescent (>6 weeks) animals with the same intensity and exposure regime, but for 30 consecutive days also leads to decreased performance in the Morris water maze [60]. However another study showed that constant 10 Ghz exposure in adolescent mice (>4 weeks) at 13 dBm (20 mW) for 6 consecutive days modulated at 8 Hz (within the theta-alpha EEG frequency band) but not at 2 Hz (within the delta EEG frequency band) decreased the spontaneous locomotor behavior in an open-field test. Despite the modulation (assuming 100% amplitude modulation), the effective microwave power (based on the root man square) used in this study and those mentioned in the studies before, are >12× higher (taking the low duty cycle of our sensors into account). Therefore we consider the microwave radiation emitted from the sensor modules used in our design to be nonhazardous for mice.

## Conclusion

We have successfully developed a simple, yet powerful open-source tool which aids laboratory practice while reducing costs. It is suitable for the beginner (e.g undergraduate behavioral neuroscience course) but holds enough expandability to satisfy the advanced. Do-it-yourself (DIY) solutions have been considered all to often as a compromise and inferior in performance compared to commercial products. However, knowing the limitations of an own design allows the careful and responsible interpretation of the obtained data, which might sometimes be better than simply relying entirely on the output of an expensive setup.

## Additional file

Additional file 1 Listings. (PDF 69 kb)

## Abbreviations

ADC: Analog-to-digital converter
ADHD: Attention deficit hyperactivity disorder
ANOVA: Analysis of variance
DIY: Do-it-yourself
EEG: Electroencephalography
EIRP: Effective isotropic radiated power
GND: Ground
GPIO: General purpose input/outputs
IDE: Integrated development environment
IGL: Intergeniculate leaflet of the the thalamus
ipRGC: Intrinsically photosensitive retinal ganglion cell
IVC: Individually ventialted cages
LAB: Low anxiety-related behavior (mice)
LCS: Light cycle shift
LED: Light-emitting diode
MDS: Motion detector shield
PCB: Printed circuit board
Pde6b: Phosphodiesterase 6b
PIR: Passive infrared
rd1: Retinal degeneration 1
RGC: Retinal ganglion cell
RHT: Retinohypothalamic tract
RTC: Real-time clock
SCN: Suprachiasmatic nucleus
SMD: Surface-mounted device
