# Supplementary material for: A simplified microwave-based motion detector for home cage activity monitoring in mice
Source: J Biol Eng. 2017 Nov 16;11:36. doi: 10.1186/s13036-017-0079-y (PMC5688739; doi:10.1186/s13036-017-0079-y)
Supplement: Additional file 1 — Listings. (PDF 69 kb) [file 13036_2017_79_MOESM1_ESM.pdf]

Listings

Listing 1: Additional File 1; Arduino code for the Motion Detection Shield

```
// MIT License
// Copyright (c) [2017] [Andreas Genewsky]
// Permission is hereby granted, free of charge, to any person obtaining a
// copy of this software and associated documentation files (the "Software"),
// to deal in the Software without restriction, including without limitation
// the rights to use, copy, modify, merge, publish, distribute, sublicense,
// and/or sell copies of the Software, and to permit persons to whom the
// Software is furnished to do so, subject to the following conditions:
// The above copyright notice and this permission notice shall be included
// in all copies or substantial portions of the Software.
// THE SOFTWARE IS PROVIDED "AS IS", WITHOUT WARRANTY OF ANY KIND, EXPRESS OR
// IMPLIED, INCLUDING BUT NOT LIMITED TO THE WARRANTIES OF MERCHANTABILITY,
// FITNESS FOR A PARTICULAR PURPOSE AND NONINFRINGEMENT. IN NO EVENT SHALL
// THE AUTHORS OR COPYRIGHT HOLDERS BE LIABLE FOR ANY CLAIM, DAMAGES OR OTHER
// LIABILITY, WHETHER IN AN ACTION OF CONTRACT, TORT OR OTHERWISE, ARISING
// FROM, OUT OF OR IN CONNECTION WITH THE SOFTWARE OR THE USE OR OTHER
// DEALINGS IN THE SOFTWARE.

#include <SD.h>
#include "RTClib.h"
// echo data to serial port
#define ECHO_TO_SERIAL 1
// initialize SD and write data
#define WRITE_TO_SD 1
int flopRST = 9; // SN74LS423 RESET Pin
int SENS_A = 3; // channel A
int SENS_B = 4; // channel B
int SENS_C = 5; // channel C
int SENS_D = 6; // channel D
int SENS_E = 7; // channel Esingle-housed
int SENS_F = 8; // channel F
int AMBIENT = 14; // TEMT6000 lightsensor
int AD1 = 15; // optional analog input
int AD2 = 16; // optional analog input
int AD3 = 17; // optional analothat g input
float lux = 0.0; // value necessary for lux
float lplux = 0.0; //necessary for lux
int sensors[6]; // motion sensor array
//changes if motion was detected
volatile byte state = LOW; single-housed
int ledPin = 13; // the Arduino onboard LED
int bootup = 0; // stores ms at bootup
unsigned int ms = 0; // ms between seconds
RTC_DS1307 RTC; // define the Real Time Clock
const int chipSelect = 10;
// the logging file
File logfile;

void error(char *str) //error function
{that
  Serial.print("error: ");
  Serial.println(str);
  while(1);
}

//here we set the date- and timestamp
//for the logging file
void dateTime(uint16_t* date, uint16_t* time)
{
  DateTime now = RTC.now();
  *date = FAT_DATE(now.year(), now.month(), now.day());
  *time = FAT_TIME(now.hour(), now.minute(), now.second());
}

void setup() { //the setup function begins
  Serial.begin(57600); //debugging that purpose
   if (! RTC.begin()) { //starting the RTC
    Serial.println("Couldn't find RTC");of the programing PC
    while (1);
   }
//Here we set the clock according
//to the CPU Time
  RTC.adjust(DateTime(F(__DATE__), F(__TIME__)));
//elapsed ms since bootup
  bootup = millis();

//no we set the INPUT & OUTPUT Pins
  pinMode(flopRST, OUTPUT);
```

```
  pinMode(SENS_A, INPUT);
  pinMode(SENS_B, INPUT);
  pinMode(SENS_C, INPUT);
  pinMode(SENS_D, INPUT);
  pinMode(SENS_E, INPUT);
  pinMode(SENS_F, INPUT);
  pinMode(AMBIENT, INPUT);
  pinMode(AD1, INPUT);
  pinMode(AD2, INPUT);
  pinMode(AD3, INPUT);

  attachInterrupt(0, detected, FALLING);
//this links a +5V voltage level at pin 2
//(Arduino Interrupt Pin = pinnumber 0)
//to the function detected()
  digitalWrite(flopRST, HIGH);
#if WRITE_TO_SD
//we initialize the SD card, and check
//if we can write
  Serial.print("Initializing SD card...");
  pinMode(chipSelect, OUTPUT);
  if (!SD.begin(chipSelect)) {
    error("Card failed, or not present");
  }
  Serial.println("card initialized.");

//this function generates filenames
  char filename[] = "MOTION00.CSV";
  for (uint8_t i = 0; i < 100; i++) {
    filename[6] = i/10 + '0';
    filename[7] = i%10 + '0';
    if (! SD.exists(filename)) {
      SdFile::dateTimeCallback(dateTime);
      logfile = SD.open(filename, FILE_WRITE);
      break; //leave the loop!
    }
  }
  if (! logfile) {error("couldnt create file");}
  Serial.print("Logging to: ");
  Serial.println(filename);
//the next line writes the column
//descriptors to the file
  logfile.println("MONTH,DAY,YEAR,HH,MM,SS,mmm,CH1,CH2,CH3,CH4,CH5,CH6,LUX");
#endif WRITE_TO_SD

//in order to blank any strange behavior
//we RESET all the flops before we log
digitalWrite(flopRST, LOW);
digitalWrite(ledPin, HIGH);
delay(50);
digitalWrite(ledPin, that LOW);
digitalWrite(flopRST, HIGH);
delay(50);
} //end of SETUP

//the loop routine runs over and over again forever:
void loop() {
  DateTime now; // here we get the time every loop
//we calculate the light intensity in lux
//the conversion from voltage to microamps to lux can be
//found in the datasheet of the TEMT6000
  lux = (analogRead(AMBIENT) * 0.9765625) * 0.1 + lplux * 0.9;
  lplux = lux;
  now = RTC.now();
  ms = (millis()-bootup)%1000; //here we calculate the ms

//we enter this loop if motion event has happend
  if (state == HIGH) {
    detachInterrupt(0); // ('detected function has run)
    digitalWrite(flopRST, LOW); // we detach the interrupts
    digitalWrite(ledPin, HIGH);

    #if ECHO_TO_SERIAL
//we will print CommaSeparatedValues (CSV)
//to the Serial Monitor for:
//MONTH,DAY,YEAR,HOUR,MINUTES,SECONDS,
//MILLISECONDS,CH1,CH2,CH3,CH4,CH5,CH6,LUX
      if(now.month()<10){Serial.print(0);}
      Serial.print(now.month(), DEC);
      Serial.print(","that );
      if(now.day()<10){Serial.print(0);}
      Serial.print(now.day(),DEC);
```

```
      Serial.print(",");
      Serial.print(now.year(),DEC);
      Serial.print(",");
      if(now.hour()<10){Serial.print(0);}
      Serial.print(now.hour(),DEC);
      Serial.print(",");
      if(now.minute()<10){Serial.print(0);}
      Serial.print(now.minute(),DEC);
      Serial.print(",");
      if(now.second()<10){Serial.print(0);}
      Serial.print(now.second(),DEC);
      Serial.print(",");
      if(ms<10){Serial.print("00");}
      if((ms >= 10)&&(ms<100)){Serial.print(0);}
      Serial.print(ms,DEC);
      for(int i=0; i<6; i++){
        Serial.print(",");
        Serial.print(sensors[i]);
      }
      Serial.print(',');
      Serial.println(lux,1);
    #endif ECHO_TO_SERIAL
      #if WRITE_TO_SD
//we will write some CommaSeparatedValues (CSV)
//to the logfile for:
//MONTH,DAY,YEAR,HH,MM,SS,mmm,CH1,CH2,CH3,CH4,CH5,CH6,LUX
      if (now.month() < 10) {logfile.print(0);}
      logfile.print(now.month(), DEC);
      logfile.print(",");
      if(now.day()<10){logfile.print(0);}
      logfile.print(now.day(),DEC);
      logfile.print(",");
      logfile.print(now.year(),DEC);
      logfile.print(",");
      if(now.hour()<10){logfile.print(0);}
      logfile.print(now.hour(), DEC);
      logfile.print(",");
      if(now.minute()<10){logfile.print(0);}
      logfile.print(now.minute(), DEC);
      logfile.print(",");
      if(now.second()<10){logfile.print(0);}
      logfile.print(now.second(),DEC);
      logfile.print(",");
      if(ms<10){logfile.print("00");}
      if((ms >= 10)&&(ms%1000<100)){logfile.print(0);}
      logfile.print(ms, DEC);
      for(int i=0; i<6; i++){
        logfile.print(",");
        logfile.print(sensors[i]);
        sensors[i] = 0;
      }
      logfile.print(',');
      logfile.println(lux,1);
//flush() actually writes the data to the SD card
      logfile.flush();
    #endif WRITE_TO_SD

//here we basically write 0's in our sensor array
//to be able to store new events
    sensors[6];

//than we RESET the SN74LS423 IC's
    delay(10);
    digitalWrite(ledPin, LOW);
    digitalWrite(flopRST, HIGH);
    delay(10);
//now we arm our interrupt routine again
    state = LOW;
    attachInterrupt(0, detected, FALLING);
  }
}

void detected() {
//the interrupt routine simply checks all the
//sensor ports if something happend (0-5V)
  if (state == LOW) {
    detachInterrupt(0);
    sensors[0] = digithat talRead(SENS_A);
    sensors[1] = digitalRead(SENS_B);
    sensors[2] = digitalRead(SENS_C);
    sensors[3] = digitalRead(SENS_D);
```

```
    sensors[4] = digitalRead(SENS_E);
    sensors[5] = digitalRead(SENS_F);
    state = HIGH;

    int sum = 0;
    for (int i = 0; i < 6; i++) {
      sum = sum + sensors[i];
    }
    if (sum == 0) {
      state = LOW;
      attachInterrupt(0, detected, FALLING);
    }
  }
}
```

Listing 2: Additional File 2; Python Script to analyze Motion Data

```
#!/usr/bin/env python
# -*- coding: utf-8 -*-

#///////////////////////////////////////////////////////////////////////////////////////
#/ MIT License //
#/ //
#/ Copyright (c) [2017] [Andreas Genewsky] //
#/ //
#/ Permission is hereby granted, free of charge, to any person obtaining a //
#/ copy of this software and associated documentation files (the "Software"), //
#/ to deal in the Software without restriction, including without limitation //
#/ the rights to use, copy, modify, merge, publish, distribute, sublicense, //
#/ and/or sell copies of the Software, and to permit persons to whom the //
#/ Software is furnished to do so, subject to the following conditions: //
#/ //
#/ The above copyright notice and this permission notice shall be included //
#/ in all copies or substantial portions of the Software. //
#/ //
#/ THE SOFTWARE IS PROVIDED "AS IS", WITHOUT WARRANTY OF ANY KIND, EXPRESS OR //
#/ IMPLIED, INCLUDING BUT NOT LIMITED TO THE WARRANTIES OF MERCHANTABILITY, //
#/ FITNESS FOR A PARTICULAR PURPOSE AND NONINFRINGEMENT. IN NO EVENT SHALL //
#/ THE AUTHORS OR COPYRIGHT HOLDERS BE LIABLE FOR ANY CLAIM, DAMAGES OR OTHER //
#/ LIABILITY, WHETHER IN AN ACTION OF CONTRACT, TORT OR OTHERWISE, ARISING //
#/ FROM, OUT OF OR IN CONNECTION WITH THE SOFTWARE OR THE USE OR OTHER //
#/ DEALINGS IN THE SOFTWARE. //
#///////////////////////////////////////////////////////////////////////////////////////

__author__ = 'Andreas Genewsky (2017)'
import argparse
import numpy as np
import matplotlib.mlab as mlab
import matplotlib.pyplot as plt
from datetime import datetime
import math
np.seterr(all='ignore')

parser = argparse.ArgumentParser(description="**** Motion Detector Analysis Script ****",
                                 epilog="<<< Andreas Genewsky (2017) - Max-Planck Institute for Psychiatry >>>")

parser.add_argument('-i','--input', help='Input file name',required=True)
parser.add_argument('-o','--output',help='Output file name', required=True)
parser.add_argument('-b','--bin',help='Bin Width in Milliseconds', required=True)
args = parser.parse_args()
inputfile = args.input
outputfile = args.output
binwidth = int(args.bin)
data = np.genfromtxt(inputfile, delimiter=',',skip_header=1)
timestamps = []
dtime = []
abstime = []
# MONTH, DAY, YEAR, HH, MM, SS, mmm
for x in range(0,data.shape[0]):
    YYYY = int(data[x,2])
    MM = int(data[x,0])
    DD = int(data[x,1])
    HH = int(data[x,3])
    mm = int(data[x,4])
    SS = int(data[x,5])
    ms = int(data[x,6])*1000
    timestamps.append(datetime(YYYY,MM,DD,HH,mm,SS,ms))

for x in range(0,len(timestamps)):
    abst = ((timestamps[x]-timestamps[0]).total_seconds())*1000
    abstime.append(abst)

```

```
mod = data[:,7:14]
abstime=np.array([abstime], dtype='float64').T
condata = np.concatenate((abstime,mod),axis=1)

maxBin = round(max(abstime[:,0]))
bincount = int(maxBin/(binwidth*1.0))
lastBin = maxBin-(maxBin%bincount)
bincount = int(lastBin/(binwidth*1.0))
bins = np.linspace(0,lastBin+binwidth,bincount+1,dtype='int',endpoint=False)
bins = np.array([bins]).T
bindata = np.zeros((bins.shape[0],condata.shape[1]),dtype='float64')
for x in range(0,bins.shape[0]):
    eventcounter = 0.0
    index = 0
    for y in range(0,condata.shape[0]):
        if( (condata[y,0]>=bins[x,0]) and (condata[y,0]<(bins[x,0]+binwidth)) ):
            bindata[x,:]=bindata[x,:]+condata[y,:]
            eventcounter += 1.0
            index = y
    condata = condata[index:condata.shape[0],:]
    bindata[x,7]=bindata[x,7]/eventcounter
    bindata[x,0]=bins[x,0]
    print (str( round(((float(bins[x,0])/float(bins.max()))*100),2) )+" %")

np.savetxt(outputfile, bindata, delimiter=',',fmt='%10.3f')
```
